# Clinicopathologic and genetic analysis of invasive breast carcinomas in women with germline *CHEK2* variants

**DOI:** 10.1007/s10549-023-07176-8

**Published:** 2023-12-13

**Authors:** Christopher J. Schwartz, Nikka Khorsandi, Amie Blanco, Rita A. Mukhtar, Yunn-Yi Chen, Gregor Krings

**Affiliations:** 1 Department of Pathology, University of California San Francisco (UCSF), 1825 4th Street, San Francisco, CA 94143 USA; 2 Department of Cancer Genetics and Prevention Program, UCSF, San Francisco, CA USA; 3 Department of Surgery, UCSF, San Francisco, CA USA

**Keywords:** *CHEK2*, Estrogen receptor, Hereditary, Breast cancer, Genomics, Neoadjuvant

## Abstract

**Purpose:**

Germline pathogenic variants in checkpoint kinase 2 (*CHEK2*) are associated with a moderately increased risk of breast cancer (BC). The spectrum of clinicopathologic features and genetics of these tumors has not been fully established.

**Methods:**

We characterized the histopathologic and clinicopathologic features of 44 *CHEK2*-associated BCs from 35 women, and assessed responses to neoadjuvant chemotherapy. A subset of cases (n = 23) was additionally analyzed using targeted next-generation DNA sequencing (NGS).

**Results:**

Most (94%, 33/35) patients were heterozygous carriers for germline *CHEK2* variants, and 40% had the c.1100delC allele. Two patients were homozygous, and five had additional germline pathogenic variants in *ATM* (2), *PALB2* (1), *RAD50* (1), or *MUTYH* (1). *CHEK2*-associated BCs occurred in younger women (median age 45 years, range 25–75) and were often multifocal (20%) or bilateral (11%). Most (86%, 38/44) were invasive ductal carcinomas of no special type (IDC-NST). Almost all (95%, 41/43) BCs were ER + (79% ER + HER2-, 16% ER + HER2 + , 5% ER-HER2 +), and most (69%) were luminal B. Nottingham grade, proliferation index, and results of multiparametric molecular testing were heterogeneous. Biallelic *CHEK2* alteration with loss of heterozygosity was identified in most BCs (57%, 13/23) by NGS. Additional recurrent alterations included *GATA3* (26%), *PIK3CA* (226%), *CCND1* (22%), *FGFR1* (22%), *ERBB*2 (17%), *ZNF703* (17%), *TP53* (9%), and *PPM1D* (9%), among others. Responses to neoadjuvant chemotherapy were variable, but few patients (21%, 3/14) achieved pathologic complete response. Most patients (85%) were without evidence of disease at time of study (n = 34). Five patients (15%) developed distant metastasis, and one (3%) died (mean follow-up 50 months).

**Conclusion:**

Almost all *CHEK2*-associated BCs were ER + IDC-NST, with most classified as luminal B with or without HER2 overexpression. NGS supported the luminal-like phenotype and confirmed *CHEK2* as an oncogenic driver in the majority of cases. Responses to neoadjuvant chemotherapy were variable but mostly incomplete.

**Supplementary Information:**

The online version contains supplementary material available at 10.1007/s10549-023-07176-8.

## Introduction

Checkpoint kinase 2 (CHK2) is a serine/threonine protein kinase encoded by the *CHEK2* gene which maintains genomic stability in the setting of DNA damage [1–3]. CHK2 is activated by the ATM kinase and in turn, interacts with BRCA1, BRCA2, p53, CDC25, and other effectors to initiate DNA repair, apoptosis, and cell cycle arrest [4–6]. Germline *CHEK2* variants were first described in patients without germline *TP53* mutations who were thought to meet clinical criteria for Li-Fraumeni syndrome [7, 8], although the association with Li-Fraumeni syndrome has since been negated [9]. Germline *CHEK2* variants have now been identified in ~ 1% of the population [9], and pathogenic variants (PVs) are causally linked to multiple types of cancer, including colon cancer and breast cancer (BC), further highlighting the functional relevance of *CHEK2* in safeguarding genome integrity [3] [10]. Germline *CHEK2* PVs confer a lifetime BC risk ranging from 20 to 44%, depending on family history of BC [11–13]. Furthermore, individuals with the higher risk c.1100delC allele carry a BC risk of up to two times higher in women and ten times higher in men when compared to non-carriers [14–16]. Accordingly, clinical guidelines currently recommend enhanced BC screening in individuals with protein truncating *CHEK2* variants [17].

Investigations into the phenotype and clinical behavior of *CHEK2*-associated BCs have been limited. *CHEK2*-associated BCs tend to occur in younger women, with higher rates of bilateral disease [12], and frequently express estrogen receptor (ER) and/or progesterone receptor (PR) [12, 13]. Interestingly, in contrast to *BRCA1/2*-related cancers, *CHEK2*-associated BCs appear to lack the genetic characteristics of homologous recombination DNA repair deficiency [18, 19]. Biallelic *CHEK2* inactivation has been observed in some but not all *CHEK2*-associated BCs [19]. The precise mechanistic relationship between *CHEK2* and BC development currently remains uncertain.

In order to characterize BC that develop in the setting of germline *CHEK2* variants, we explored the clinicopathologic, histologic, and genetic features of BCs arising in a consecutive series of patients with pathogenic or likely pathogenic germline *CHEK2* variants (P/LPVs) at our institution.

## Methods

### Study population

The institutional review board of the University of California San Francisco (UCSF) approved this study. Patients with P/LPVs in *CHEK2* who underwent breast tissue sampling were retrospectively identified over a 15-year period (2008 to 2023) using a pathology database. All germline variants in this cohort (n = 35) with invasive BC were reviewed by a board-certified genetic counselor (AB) and classified as P/LPVs according to the ClinVar database [20].

### Study design

All slides were reviewed by at least one breast pathologist (CJS, YYC, and/or GK). For primary BCs, ER, PR, and human epidermal growth factor receptor 2 (HER2) were evaluated and scored according to ASCO/CAP guidelines [21, 22]. Ki67 was scored according to recommendations of the International Ki67 in Breast Cancer Working Group [23]. Immunohistochemical surrogates of molecular subtype were classified based on St. Galen criteria as previously described using either 14% or 20% cut-offs for Ki-67 [24]. Tumor-infiltrating lymphocytes (TILs) were assessed based on recommendations by the TILs Working Group [25]. For neoadjuvant chemotherapy (NACT)-treated tumors, assessment of tumor cellularity and residual cancer burden (RCB) index were calculated according to methods previously described [26]. Clinicopathologic and outcome data were retrieved from the electronic medical record.

### Targeted next-generation DNA sequencing

Tumor tissues were selected from primary BCs (n = 21), lymph node metastasis (n = 1), lung metastasis (n = 1), and liver metastasis (n = 1) for capture-based targeted next-generation DNA sequencing (NGS). Sequencing was performed at the UCSF Clinical Cancer Genomics Laboratory using an assay that targets all coding regions of ~ 500 cancer-related genes, select introns from 40 genes, and *TERT* promoter, and analyzed according to methods previously described [27].

## Results

### Study overview

**Fig. 1:**
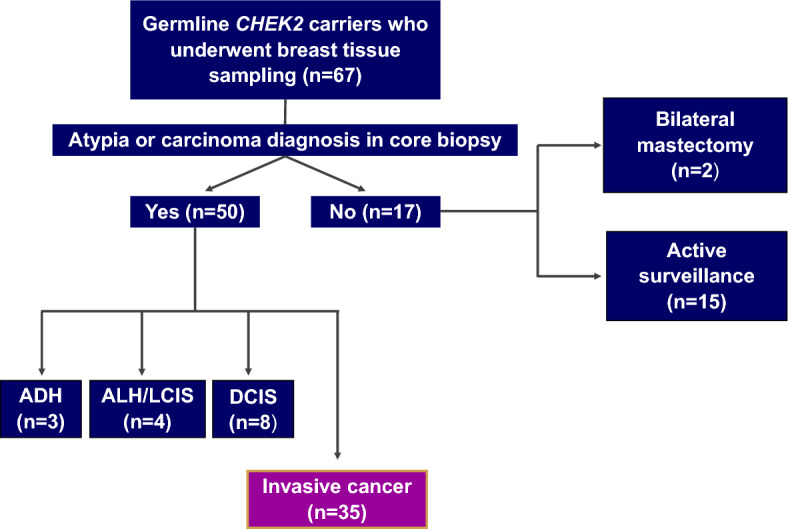
Schematic workflow for identifying breast cancers arising in individuals with *CHEK2* pathogenic and likely pathogenic variants

The workflow for this study is depicted in Fig. 1. We identified 67 patients with *CHEK2* P/LPVs who underwent core biopsy of the breast. Most patients (50/67, 75%) had intraductal epithelial atypia or in situ or invasive carcinoma on core biopsy. Of these, noninvasive disease included atypical ductal hyperplasia (4%, 3/67), lobular neoplasia (atypical lobular hyperplasia or lobular carcinoma in situ) (6%, 4/67), and ductal carcinoma in situ (12%, 8/67). Invasive BC was identified in 52% of patients (35/67), and these patients comprised the subsequent study population. In total, 44 invasive BCs were analyzed from these 35 patients. Most patients (88%, 15/17) without atypia or carcinoma on core biopsy were under active surveillance, with two patients opting for bilateral risk-reducing mastectomy (Fig. 1).

### Indications for genetic testing and description of germline *CHEK2* variants in patients with invasive breast cancer

Genetic testing followed the personal diagnosis of BC in 83% (29/35) of patients (Table 1). A smaller subset (11%, 4/35) underwent genetic testing after a germline *CHEK2* variant was identified in a family member. Most patients (79%, 27/34) had a family history of BC, with 41% (14/34) occurring in a first degree relative (Table 1). Three patients had a previous history of BC prior to presentation at our institution (9%, 3/35).

**Table 1:** Indication for genetic testing and cancer history in patients with *CHEK2*-associated invasive breast cancer

Indication for genetic testing	
After initial cancer diagnosis	29/35 (83%)
Family member with *CHEK2*	4/35 (11%)
Strong family history/desired testing	1/35 (3%)
WISDOM trial ^a^	1/35 (3%)
Family history of breast cancer^b^	27/34 (79%)
Family history of breast cancer in 1st degree relative^ab^	14/34 (41%)
Personal cancer history	6/35 (17%)

^a^Women Informed to Screen Depending on Measures of Risk Trial ^b^ One patient was adopted

A summary of the germline *CHEK2* P/LPVs in patients that developed invasive BC and associated clinical characteristics are detailed in Supplementary Table S1. C*HEK2* P/LPVs consisted of frameshift (17/35, 49%), missense (26%, 9/35), and splice site (11%, 4/35) mutations, as well as exonic deletions (14%, 5/35) (Table 2). The cohort was enriched for *CHEK2* c.1100delC (p.T367fs) variants (40%, 14/35), which is thought to portend the highest BC risk [14–16]. The second most common variant was c.1283C > T (p.S428F) (9%, 3/35), a low penetrance missense mutation thought to abrogate the kinase function of CHK2 [28]. Nearly all patients (33/35, 94%) were heterozygous *CHEK2* carriers (Table 2). Two patients (patient 2, c.1100delC and patient 32, c.499G > A) were homozygous with a family history of BC in a first-degree relative. Five patients (14%) additionally harbored PVs in other genes associated with BC risk, including *ATM* (c.1027_1030del, p.E343Ifs and c.237del, p.Lys79fs), *PALB2* (c.2827_2830del, p.E943Sfs), *RAD50* (c.2517dupA, p.D480fs) and *MUTYH* (c.536A > G, p.Y179C) (Supplementary Table S1).

**Table 2:** Germline pathogenic/likely pathogenic variants in patients with invasive breast cancer

*CHEK2* variant status	
Heterozygous	33/35 (94%)
Homozygous	2/35 (6%)
*CHEK2* variant	
Frameshift	17/35 (49%)
c.1100delC (p.T367Mfs)^a,b^	14/35 (40%)
c.1263delT (p.S422fs)	2/35 (6%)
c.433delC (p.R145fs)^c^	1/35 (3%)
Missense	9/35 (26%)
c.1283C > T (p.S428F)^a^	3/35 (9%)
c.470 T > C (p.I157T)	2/35 (6%)
c.349A > G (p.R117G)	2/35 (6%)
c.499G > A (p.G167R)	1/35 (3%)
c.707 T > C (p.L236P)^d^	1/35 (3%)
Exon deletions	5/35 (14%)
Splice site	4/35 (11%)

^a^One patient with germline pathogenic/likely pathogenic *ATM* variant

^b^One patient with germline pathogenic/likely pathogenic *PALB2* variant

^c^One patient with germline pathogenic/likely pathogenic *MUTHY* variant

^d^One patient with germline pathogenic/likely pathogenic *RAD50* variant

### Clinicopathologic characteristics of *CHEK2*-associated breast cancers

The clinicopathologic features of *CHEK2*-associated invasive BCs are shown in Table 3. All patients were women. The median age at diagnosis was 45 years (range 25–75, Table 3). Most presented with a palpable mass (54%, 19/35). The remainder had either a mammographically detected mass (29%, 10/35) or calcifications (17%, 6/35). Four patients had synchronous bilateral BCs (11%, 4/35) and seven had multifocal BC (20%, 7/35). The mean tumor size in non-treated cases was 1.3 cm (range < 0.1 to 5.4) (Table 3). Lymph node metastases were present in 38% of cases (13/34).

**Table 3:** Clinicopathologic characteristics of breast cancers arising in patients with *CHEK2* pathogenic/likely pathogenic germline alterations

Median age, years (range)	45 (25–75)
Clinical presentation	
Palpable mass	19/35 (54%)
Mammographically-detected mass	10/35 (29%)
Mammographically-detected calcifications	6/35 (17%)
Mean non-treated tumor size, cm (range)	1.3 (< 0.1 to 5.4)
Bilaterality	4/35 (11%)
Multifocality	7/35 (20%)
Lymph node metastasis	13/34 (38%)
Invasive tumor type^a,b^	
Invasive ductal carcinoma^c^	38/44 (86%)
Invasive lobular carcinoma	3/44 (7%)
Invasive carcinoma with ductal and lobular features	2/44 (5%)
Papillary carcinoma	1/44 (2%)
Hormone receptor profile	
ER +	41/43 (95%)
ER + HER2−^d^	34/43 (79%)
ER + HER2 +	7/43 (16%)
ER−	2/43 (5%)
ER-HER2−	0/43 (0%)
ER-HER2 +	2/43 (5%)
Ki67^e^	
≤ 5%	3/31 (10%)
6–29%	18/31 (58%)
≥ 30%	10/31 (32%)
Molecular subtype	
Luminal A	9/36 (25%)
Luminal B	25/36 (69%)
HER2-positive (non-luminal)	2/36 (6%)
Basal-like	0/36 (0%)
Nottingham grade‡	
1	8/44 (18%)
2	25/44 (57%)
3	11/44 (25%)
OncotypeDX	
< 17 (Low)	5/12 (42%)
18–30 (Intermediate)	5/12 (42%)
> 31 (High)	2/12 (16%)
MammaPrint	
Low-risk	6/13 (46%)
High-risk	7/13 (54%)

^a^Three patients had multiple synchronous ipsilateral tumors

^b^Four patients had multiple synchronous bilateral tumors

^c^One patient had invasive ductal carcinoma with minor apocrine component

^d^One patient had an ER + HER2 + metachronous metastasis

^e^Pre-treatment Ki67 for treated tumors

^‡^Pre-treatment histologic grade

BCs were predominantly invasive ductal carcinomas of no special type (IDC-NST) (86%, 38/44). Other histologic types were invasive lobular carcinoma (7%, 3/44, including solid [1], alveolar [1], and classic [1] patterns), invasive carcinoma with ductal and lobular features (5%, 2/44), and invasive papillary carcinoma (2%, 1/44). BCs were most often Nottingham grade 2 (57%, 25/44), with 25% (11/44) grade 3 and 18% (8/44) grade 1. TILs were sparse (< 10%) in most (89%, 34/38) cases and > 20% in only one case (3%, 1/34) (Supplemental Table S2).

Nearly all BCs were ER + (95%, 41/43), including 79% (34/43) ER + HER2- and 16% (7/43) ER + HER2 + . Both ER- tumors were HER2 + (5%, 2/43). Of the nine total HER2 + BCs, seven (78%) arose in a background of *CHEK2* c.1100delC, and, conversely, 43% (6/14) of BCs arising in patients with *CHEK2* c.1100delC were HER2 + . No patients developed triple negative BC. The Ki67 proliferation index was ≥ 30% in 32% (10/31), 6–29% in 58% (18/31), and ≤ 5% in 10% (3/31) of cases. Using immunohistochemical surrogates for molecular subtyping, most (69%, 25/36) BCs were classified as luminal B (50%, 18/36 Luminal B HER2-, 19%, 7/36 Luminal B HER2 + ; using ≥ 14% Ki-67 cutpoint) [24]. One-quarter (25%, 9/36) were luminal A, and only 6% (2/36) were HER2-enriched. Results were similar when using ≥ 20% Ki-67 cutpoint for Luminal B (67% luminal B) (Supplemental Table S2).

OncotypeDX scores were available for 12 patients and were variable, with most (83%) scores falling into the low (5/12) or intermediate categories (5/12). Of the two patients with high OncotypeDX scores (16%, 2/12), one developed lung metastasis. MammaPrint assay scores were available for 13 patients and stratified nearly equally into low-risk (46%, 6/13) and high-risk categories (54%, 7/13).

### Next-generation DNA sequencing

**Fig. 2:**
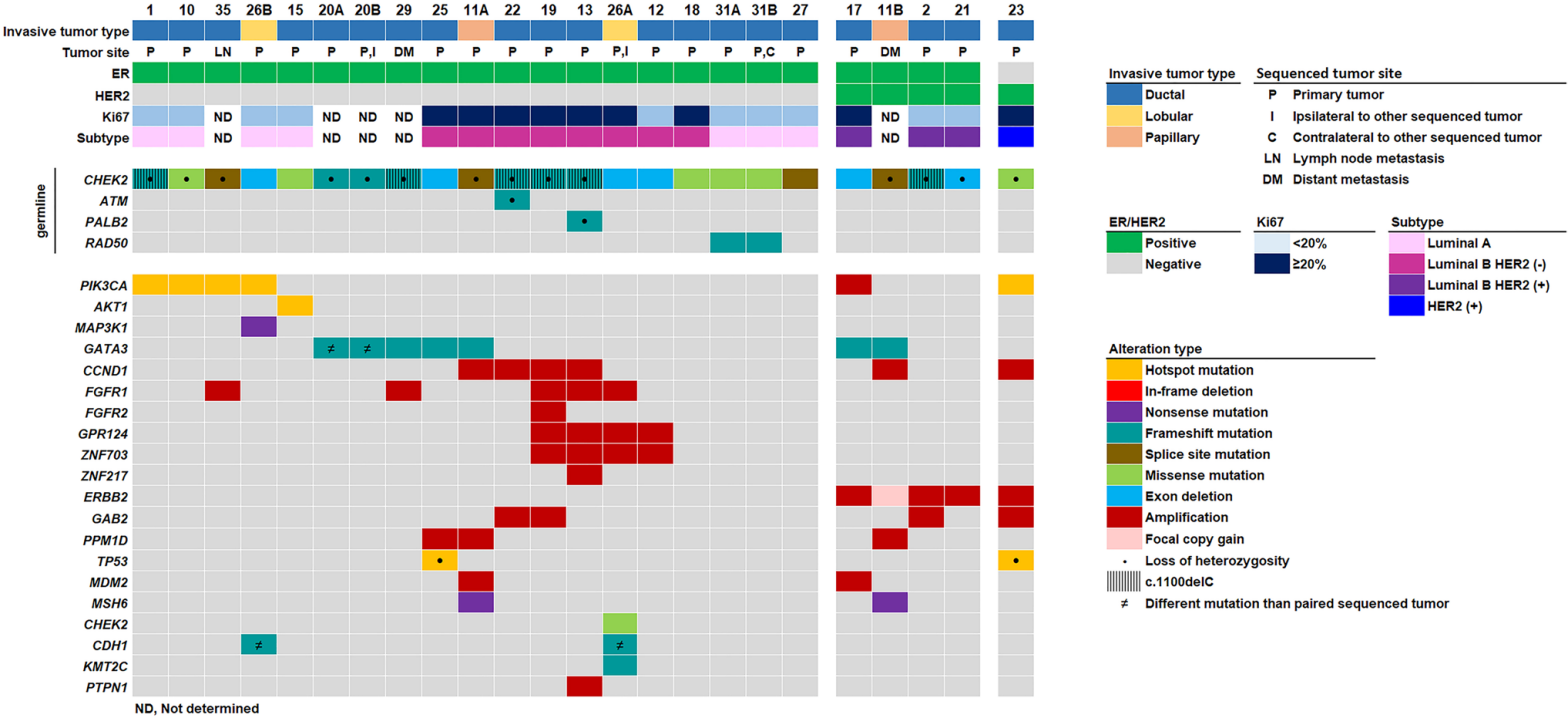
Genetic profiling of invasive breast cancers arising in patients with germline *CHEK2* variants

Targeted next-generation DNA sequencing was performed on 23 BCs (24 samples) from 20 patients, including 21 primary BCs, one lymph node metastasis,one liver metastasis, and one lung metastasis. The results are shown in Fig. 2. Sequenced primary BCs included synchronous unilateral tumors from two patients and synchronous bilateral tumors from one patient. Most (92%, 21/23) sequenced tumors were IDC-NST. All were ER + , and 22% (5/23) were HER2 + by immunohistochemistry and/or fluorescence in situ hybridization, including a metachronous ER + HER2 + lung metastasis in Patient 11.

Biallelic *CHEK2* alteration was detected in 13 of 23 BCs (57%), which was due to somatic loss of heterozygosity (LOH) of the germline *CHEK2* allele in all cases. All sequenced BCs arising in patients with germline *CHEK2* c.1100delC (n = 6) showed biallelic *CHEK2* inactivation via LOH. In one case (patient 26), an additional somatic missense *CHEK2* mutation (p.K373E) was identified in an invasive lobular carcinoma (ILC) arising in a 70-year-old germline *CHEK2* exon 9 deletion carrier. Whether this second *CHEK2* hit was in *cis* or *trans* with the germline allele could not be determined. A synchronous ipsilateral ILC in the same patient was heterozygous for the germline *CHEK2* mutation with no additional *CHEK2* alterations. The genetic profiles of the synchronous ILCs in this patient were also otherwise distinct, including different inactivating *CDH1* mutations (p.T115fs and p.N315fs) among other alterations (Fig. 2).

Aside from biallelic alteration of *CHEK2*, recurrent alterations included inactivating *GATA3* mutations (6/23, 26%), *PIK3CA* hotspot (p.H1047R) mutations or amplification (6/23, 26%), and amplifications of *CCND1* (5/23, 22%), *FGFR1* (5/23, 22%), *ERBB2 *(4/23, 17%), *GAB2* (4/23, 17%), *ZNF703* (4/23, 17%), *GPR124* (4/23, 17%), *PPM1D *(2/23, 9%), and *MDM2* (2/23, 9%). *TP53* mutation were infrequent (2/23, 9%).

Invasive BCs from 3 of the 5 patients with concurrent PVs in additional BC risk genes were sequenced (Fig. 2). Patient 13 (*CHEK2* c.1100delC and *PALB2* c.2827_2830del) presented with a 2 cm Nottingham grade 3 ER + HER2- IDC-NST at age 25 (Oncotype 21). Tumor sequencing revealed LOH in *CHEK2* and *PALB2*, as well as *CCND1*,*FGFR1*, *GPR124*, *ZNF217*, *ZNF703*, and *PTPN1* amplifications. Patient 22 (*CHEK2* c.1100delC and *ATM* c.237del) presented with a Nottingham grade 3 ER + HER2- IDC-NST at age 30 (MammaPrint high-risk). Tumor sequencing revealed LOH in *CHEK2* and *ATM*, as well as *CCND1* and *GAB2* amplifications. This patient received neoadjuvant chemotherapy and was one of only three patients to achieve pCR. Patient 31 (*CHEK2* c.707 T > C (p. Leu236Pro and *RAD50* c.2517dupA, p. D480fs) presented at age 45 with synchronous bilateral IDC-NST. No additional somatic alterations were identified on sequencing of either tumor.

### Treatment and Outcome

Treatment and outcomes of *CHEK2*-associated BCs are shown in Table 4. Most patients underwent mastectomy (69%, 24/35) compared to lumpectomy (31%, 11/35). Fourteen patients received NACT (40%, 14/35), and five patients received neoadjuvant hormonal therapy (NAHT) (14%, 5/35). In line with the high frequency of ER + BCs, nearly all (88%, 30/34) patients received adjuvant hormonal therapy. Twelve patients received adjuvant radiotherapy (35%), and 13 patients received adjuvant chemotherapy (38%).

**Table 4:** Treatment, follow-up and outcomes of patients with *CHEK2*-associated breast cancers

Surgical procedure	
Partial mastectomy^a^	11/35 (31%)
Total mastectomy	24/35 (69%)
Neoadjuvant therapy	
Chemotherapy	14/35 (40%)
Hormone therapy	5/35 (14%)
Adjuvant therapy	
Radiation	12/34 (35%)
Hormonal therapy	30/34 (88%)
Chemotherapy	13/34 (38%)
Patients with follow-up	34/35 (97%)
Mean follow-up, months (range)	50 (13–183)
Local recurrence	1/34 (3%)
Distant metastasis	5/34 (15%)
Disease status	
No evidence of disease	29/34 (85%)
Alive with disease	4/34 (12%)
Died of disease	1/34 (3%)

^a^One patient underwent completion mastectomy after 1 year

Clinical follow-up was available for 34 patients (97%, 34/35) with a mean interval of 50 months. One patient (3%, 1/34) developed local recurrence, which was an axillary recurrence of ER + IDC-NST 72 months after bilateral mastectomy. Five patients (15%) developed distant metastases, with metastatic sites including liver (2), bone (2) and lung (1) (Supplementary Table S2). The majority of patients (85%, 29/34) had no evidence of disease at time of study, whereas 12% (4/34) are alive with disease. Only one patient (3%) died of disease following a poor response to NACT, liver metastasis at 58 months, and death at 117 months. Among the 11 patients who opted for breast conservation, none developed local recurrence during the follow-up period.

### Response to neoadjuvant chemotherapy

Treatment responses to NACT of 18 *CHEK2*-associated BCs arising in 14 patients are summarized in Supplemental Table S3. All BCs (100%) showed a clinical reduction in tumor size based on tumor size at pathologic examination compared to pre-treatment magnetic resonance imaging (MRI). All BCs with available data showed a post-treatment reduction in Ki67 proliferation index compared to pre-treatment scores. By RCB calculation, most treated BCs were RCB-II (11/18, 61%) or RCB-I (4/18, 22%), with no RCB-III tumors. Pathologic complete response (pCR) was observed in only three patients (3/14, 21%), two of which had HER2 + BC. In cases with residual cancer, invasive tumor cellularity ranged from < 1% to 50% and was ≥ 30% in 50% (7/14) of tumors.

## Discussion

In summary**,** *CHEK2*-associated invasive BCs tended to arise in younger women with a strong family history of BC and were often multifocal and bilateral. The tumors were mostly immune-poor ER + IDC-NST of predominantly luminal B subtype and were heterogeneous in terms of histologic grade, HER2 status, proliferation index, and multi-parametric molecular testing. NGS overall showed luminal-like genetics and biallelic *CHEK2* alteration via LOH as an oncogenic driver in most cases. BCs arising in patients with concurrent germline PVs in the DNA damage genes *PALB2* and *ATM* (but not *RAD50*) also showed LOH in these genes. Most patients with *CHEK2*-associated BCs had partial responses to NACT, with few achieving pCR.

Most but not all *CHEK2*-associated BCs showed biallelic *CHEK2* alteration via LOH, which is in keeping with incomplete rates of LOH seen in prior studies [18, 19, 29, 30]. A recent study found high rates of *CHEK2* LOH in BCs arising in c.1100delC carriers compared to lower rates in other *CHEK2* variants [19]. Consistent with this, we found *CHEK2* LOH in all BCs arising in c.1100delC carriers (6/6) but only in 47% (8/17) of BCs arising in other variants. Taken together, our findings and those of others raise consideration that at least some *CHEK2* variants may function via haploinsufficiency in driving BC, whereas others (such as c.1100delC) may require bilallelic inactivation. This is in contrast to most other germline BC risk genes, such as *BRCA1*/*2, PALB2*, and *ATM*, PVs of which often show biallelic loss of function in BC [29]. Alternatively, CHK2 inactivation may also be due to non-genetic mechanisms in cases without LOH, and future studies may help address this issue.

Most BCs arising in our population of *CHEK2* carriers were ER + IDC-NST, predominantly of luminal B subtype, with a minority of tumors being luminal A or HER2-enriched, and none being triple negative. These findings are consistent with previous reports showing a predominance of ER/luminal BC and HER2 overexpression in this setting [9, 12, 19, 31, 32]. Aside from biallelic *CHEK2* inactivation in the majority of cases, the mutational repertoire of the sequenced luminal BCs in our series was otherwise similar to other luminal BCs and included recurrent aberrations in genes such as *PIK3CA*, *GATA3*, *CCND1*, *FGFR1*, and *ERBB2*, with a low frequency of *TP53* mutations. Our results are thus overall consistent with those of previous studies [18, 19, 31, 33–35] and highlight *CHEK2* as a dominant oncogenic driver in these tumors while also supporting a model in which *CHEK2* may act as a facilitator or accelerator of tumorigenesis that is otherwise subtype-specific. This model of *CHEK2*-facilitated tumorigenesis was previously put forth by Massink et al. in their study describing luminal-like copy number profiles of BCs arising in *CHEK2* c.1100delC carriers [18].

Concurrent germline P/LPVs in multiple hereditary BC risk genes (so-called double heterozygotes) are rare, and data regarding cancer risk and clinical implications are scarce. The majority of reported double heterozygotes in the literature include *BRCA1* and/or *BRCA2*, with only few studies and case reports describing double heterozygosity exclusively involving other BC risk genes, such as *CHEK2*, *ATM*, *PALB2*, *NBS1*, and *BLM* [36–44]. Given the rarity of these events, conclusions regarding cancer risk, cancer phenotype, patient surveillance, and clinical management of these cases are limited. It has been suggested that *BRCA1/2* and *CHEK2* double heterozygotes do not have increased BC risk or a different BC phenotype beyond what is expected for *BRCA1/2* variants [39, 40, 44]. Whether a similar principle holds for double heterozygotes involving *CHEK2* and non-*BRCA1/2* BC risk genes is not known due to paucity of data. Taken as a whole, BCs arising in double heterozygotes do not appear to present at younger age or with increased bilaterality compared to single heterozygotes [39]. Of the five double heterozygotes involving *CHEK2* with *ATM*, *PALB2*, *RAD50*, or *MUTYH* in our study, we noted a younger age of BC presentation than the cohort median in three cases (*CHEK2* c.433delC/*MUTYH* c.536A > G, age 34; *CHEK2* c.1100delC/*PALB2* c.2827_2830del, age 25; *CHEK2* c.1100delC/*ATM* c.237del, age 29), and bilateral and multifocal tumors in only one patient (*CHEK2* c.707 T > C/*RAD50* c.2517dupA). We therefore find no compelling evidence of a synergistic effect compared to *CHEK2* carriers alone, but note that the small number of such cases in our series significantly limits meaningful conclusions. There is similarly little data on BC risk and phenotype in *CHEK2* homozygotes or compound heterozygotes, although available data suggests that these patients may have higher BC risk, present at younger age, and more often have a second BC diagnosis compared to heterozygotes [45–47]. Our study included two *CHEK2* homozygotes (c.1100delC and c.499G > A), who presented with BC at 45 and 39 years of age, respectively, and have not developed additional BCs at time of study.

Ours is one of few studies assessing the response of *CHEK2*-associated BCs to NACT. Although decreased post-treatment tumor size and Ki67 proliferative index reflected treatment effect in all evaluable cases, only three patients in our series achieved pCR. Two tumors with pCR were HER2 + (Luminal B HER2 + and HER2 enriched). These results are not surprising, given the overall lower pCR rates of ER + /luminal tumors [48, 49] as well as the absence of robust homologous recombination DNA repair deficiency in *CHEK2*-associated BC [18, 19, 30]. Indeed, our results are similar to a prior study of poor NACT responses in eight *CHEK2* patients with BC, in which responses to NACT were also found to be worse in *CHEK2* carriers when compared to non-carriers [49]. Larger studies are required to confidently compare NACT sensitivities of *CHEK2*-associated ER + BCs to other BCs. We speculate that the absence of significant immune cell infiltration may also impact response rates to NACT.

Guidelines recommend annual mammography with consideration for magnetic resonance imaging starting at age 40 in patients with *CHEK2* P/LPVs. However, unlike for patients with germline *BRCA1* or *BRCA2* alterations, there are currently no guidelines regarding risk-reducing mastectomy in *CHEK2* carriers, and decision making is generally based on individualized and family risk [17]. There is a paucity of data evaluating recurrence rates after breast conservation versus unilateral or bilateral mastectomy in *CHEK2* carriers with BC, although some studies have suggested a higher risk of contralateral BC [32, 50, 51]. In our study, only 11 patients with BC opted for breast conservation, and none experienced local recurrence. On the other hand, we found higher than expected rates of bilateral and multifocal tumors compared to the general population, which is consistent with prior studies [52] and has implications arguing against breast conservation. Aside from *CHEK2* germline status and type of *CHEK2* variant (*i.e.* c.1100delC vs other), patient age at presentation, family history, and patient preferences, should also be considered when planning surgical intervention and surveillance for these patients.

### Supplementary Information

Below is the link to the electronic supplementary material.

Supplementary file1 (XLSX 41 KB)

Supplementary file2 (XLSX 41 KB)

## Data Availability

Inquiries about data availability should be directed to the authors.
